# The Nutritional Status of Long-Term Institutionalized Older Adults Is Associated with Functional Status, Physical Performance and Activity, and Frailty

**DOI:** 10.3390/nu13113716

**Published:** 2021-10-22

**Authors:** Itxaso Mugica-Errazquin, Idoia Zarrazquin, Jesús Seco-Calvo, Javier Gil-Goikouria, Ana Rodriguez-Larrad, Janire Virgala, Nagore Arizaga, Beatriz Matilla-Alejos, Jon Irazusta, Maider Kortajarena

**Affiliations:** 1 Department of Nursing II, Faculty of Medicine and Nursing, University of the Basque Country, Paseo Doctor Begiristain 107, 20014 Donostia-San Sebastián, Spain; itxaso.mugica@ehu.eus (I.M.-E.); idoia.zarrazquin@ehu.eus (I.Z.); 2 Osakidetza Basque Health Service, General Surgery Service, Donostia University Hospital, Paseo Doctor Beguiristain, 20014 Donostia-San Sebastian, Spain; 3 Institute of Biomedicine (IBIOMED), Campus of Vegazana, University of León, 24071 León, Spain; jesus.seco@unileon.es; 4 Department of Physiology, Faculty of Medicine and Nursing, University of the Basque Country, Bº Sarriena s/n, 48940 Leioa, Spain; javier.gilgoikouria@ehu.eus (J.G.-G.); ana.rodriguez@ehu.eus (A.R.-L.); janire.virgala@ehu.eus (J.V.); nagore.arizaga@ehu.eus (N.A.); jon.irazusta@ehu.eus (J.I.); 5 Osakidetza Basque Health Service, OSI Tolosaldea, Tolosa Primary Care Center, 20400 Tolosa, Spain; 6 Osakidetza Basque Health Service, OSI Donostialdea, Zarautz Primary Care Center, 20800 Zarautz, Spain; 7 Santa Maria Magdalena Long-Term Nursing Home, Sandiusterri 1, 20120 Hernani, Spain; lahar@hernani.eus

**Keywords:** nutritional status, functional status, physical performance, physical activity, frailty, older adults, long-term institutionalization

## Abstract

Among older adults living in long-term nursing homes (LTNHs), maintaining an adequate functional status and independence is a challenge. Whilst a poor nutritional status is a potential risk factor for a decreased function in this population, its role is not fully understood. Here, using a transversal multicenter study of 105 older adults living in 13 LTNHs, we analyzed the associations between nutritional status, as measured by the Mini Nutritional Assessment (MNA), and the parameters of functional status, physical performance, physical activity, and frailty as well as comorbidity and body composition. The MNA scores were positively correlated with the Barthel Index, handgrip strength, Short Physical Performance Battery (SPPB) scores, absolute muscle power, and Assessment of Physical Activity in Frail Older People (APAFOP) scores and were negatively correlated with dynamic balance and frailty. In a multiple linear regression model controlling for gender and age, the APAFOP score (β = 0.386), BMI (β = 0.301), and Barthel Index (β = 0.220) explained 31% of the variance in the MNA score. Given the observed close relationship between the MNA score and functional status, physical performance and activity, and frailty, interventions should jointly target improvements in both the nutritional status and functional status of LTNH residents. Strategies designed and implemented by interdisciplinary professional teams may be the most successful in improving these parameters to lead to better health and quality of life.

## 1. Introduction

Given the aging of the global population, maintaining an adequate functional status and independence among older adults is a fundamental challenge. Although recent years have seen much progress in this area with extensive research in the field of frailty and quality of life in older individuals, many issues remain unresolved such as clear evidence of the effect of physical activity or nutrition in slowing the progression of frailty or functional deterioration in aging especially in older adults living in long-term nursing homes (LTNH). In particular, the role that nutritional status may play in functional status, physical performance and activity, and frailty remains unclear.

According to the International Association of Gerontology and Geriatrics and the International Academy of Nutrition and Aging, the risk factors for malnutrition in older adults include being 85 years or older, a low nutrient intake due to a loss of the ability to eat independently, difficulty swallowing and chewing, becoming bedridden, pressure ulcers, a history of hip fracture, dementia, depressive symptoms, and suffering from two or more chronic illnesses [1,2,3,4]. In older adults living in LTNHs, major risk factors include mobility limitations and a higher age; in community-dwelling older adults, such factors include a poor appetite, difficulties with eating, and respiratory and gastrointestinal diseases [5].

Several risk factors such as a lack of the ability to eat independently, being bedridden, pressure ulcers related to immobility, hip fractures, and even depressive conditions can be countered or prevented by physical activity [6,7,8,9,10,11,12]. In frail older adults living in the community, physical exercise improves the physical, psychological, and social capacity and reduces the risk of falls and of losing independence [13,14]. In contrast, the few studies that have focused on the residents of nursing homes vary widely both in participant characteristics and in the interventions implemented, making it difficult to draw conclusions to inform best practices. Older individuals living in LTNHs generally have a sedentary lifestyle with very few minutes per day of physical activity performed mainly at a low intensity. This inactivity both leads to a loss of the musculoskeletal function and reduces opportunities to engage in experiences that improve the quality of life [15].

Institutionalized and community-dwelling older populations differ in their body composition, nutritional deficiencies, predisposition to malnutrition, cognitive impairment, and deterioration in physical performance [5,16]. Whilst undernutrition is associated with a decreased physical performance and with a disability in community-dwelling older adults [17,18,19,20], the role of the nutritional status of older adults living in LTNHs is less clear. In one study of nursing home residents, mobility and ADL dependency were impaired in older adults with (a risk of) malnutrition [21]. In this regard, Ramsey et al. observed that malnutrition was also linked to a lower physical performance in a dynamic test in geriatric outpatients [22]. In contrast, in a previous study on a group of older adults residing in senior living facilities, a 6-month exercise and nutritional supplement program did not improve the physical fitness or activity levels due to several facility characteristics that hindered the implementation of the program [23]. Such findings indicate the importance of developing further studies involving older adults living in LTNHs and analyzing the role that nutrition might play in parameters such as functional status, physical performance and activity, and frailty.

Malnutrition may also increase the risk of frailty. Two recent reviews highlighted the relative lack of studies conducted on frail patients, particularly the lack of nutritional intervention studies especially clinical trials [24,25]. Reflecting this lack, two of the few clinical trials with a nutritional intervention performed in frail older individuals living in LTNHs are the one published by Fiatarone et al. and Smoliner et al. [26,27]. The nutritional programs they conducted involved LTNH residents at a risk of malnutrition and the duration of them was 10 and 12 weeks, respectively. Fiatarone et al. supplemented with a multinutrient liquid supplement and Smoliner et al. with fortified food. Although the nutritional status improved in both studies, neither of them observed physical performance or functional gains associated with supplementation.

Thus, despite evidence linking nutritional status with physical and functional status of the LTNH population, its effect on these variables remains unclear. Here, we analyzed the relationship between the nutritional status, as measured by the Mini Nutritional Assessment (MNA), and functional status, physical fitness and activity, and frailty in 105 older adults living in 13 LTNHs. Understanding this relationship can help inform strategies to promote health and increase the quality of life among institutionalized older adults.

## 2. Materials and Methods

### 2.1. Study Design and Participants

**Figure 1:**
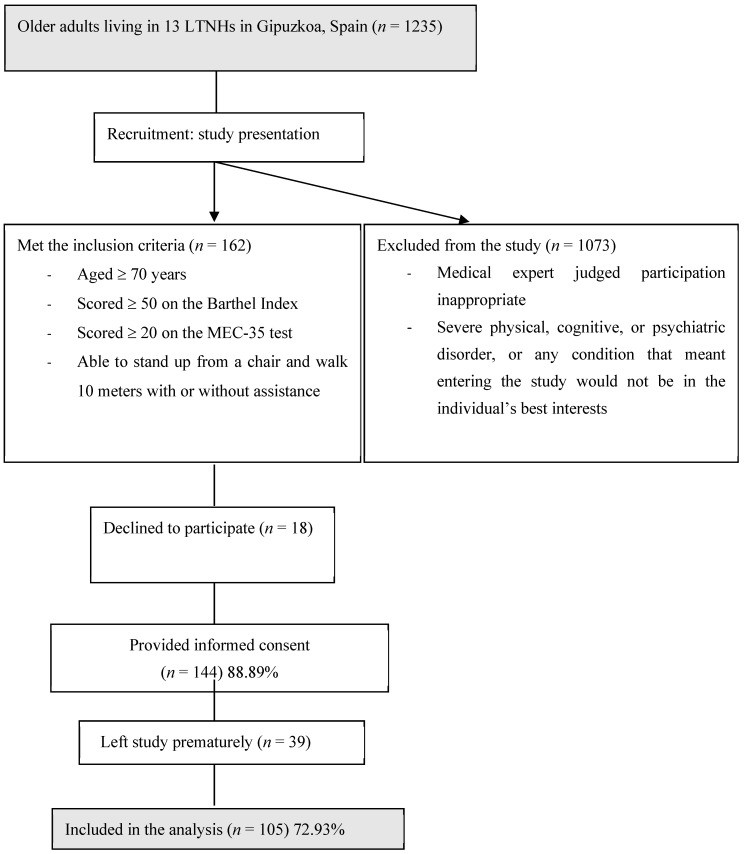
Study flow diagram.

This transverse, multicenter study was a secondary analysis of a clinical trial of a multicomponent physical exercise program (registry number NCT04221724) of LTNH residents. Data were collected between September and December 2019 in the post-intervention stage. The nutritional status, body composition, functional status, physical performance, physical activity, and frailty parameters were measured. Each parameter was measured by the same researcher to avoid an inter-assessor bias. The participants were recruited from 13 LTNHs in Gipuzkoa (Basque Country, Northern Spain). The inclusion criteria were as follows: aged ≥ 70 years, scored ≥ 50 on the Barthel Index, scored ≥ 20 on the Mini Examen Cognoscitivo (MEC-35) test [28], and able to stand up from a chair and walk 10 m with or without assistance. Individuals were not eligible to be included in the study if participation was judged to be inappropriate by a medical expert due to any risk of heart failure or ischemic events, e.g., in cases of severe physical, cognitive, or psychiatric disorders or in cases of any other condition such that participation would not be in the best interests of the individual. After considering these criteria, 105 participants were included in the study (Figure 1). All participants provided informed consent and the Committee on Ethics in Research of the Basque Country University (Human Committee Code M10/2018/171) approved the study.

### 2.2. Variables Measured

The nutritional status was assessed using the full version of the MNA [29]. The MNA scores ranged from 0 to 30 with a status classified as normal nutrition (24–30 points), a potential risk of malnutrition (17–23.5 points), and malnutrition (<17 points).

The clinical data were collected from the databases of the participating LTNHs. The comorbidities were calculated and categorized according to an age-adjusted Charlson comorbidity index, where one point was added for each decade after the age of 50 years [30].

The anthropometric measures included height, weight, and body mass index (BMI) (kg·m^−2^). The BMI was classified according to the National Heart, Lung and Blood Institute guidelines: underweight (<18.5 kg/m^2^), desirable (18.5–24.9 kg/m^2^), overweight (25.0–29.9 kg/m^2^), obesity (30.0–39.9 kg/m^2^) and morbid obesity (≥40 kg/m^2^) [31].

The functional status was assessed by the Barthel Index [32], which measured 10 activities of daily living (ADL) (bowels, bladder, grooming, toilet use, feeding, transfer, mobility, dressing, stairs, bathing). The index ranged from 0 to 100 with 100 indicating complete independence. The assessment was carried out with the help of a caregiver.

The physical performance was assessed using a Short Physical Performance Battery (SPPB), the best-known instrument for evaluating the physical performance of senior citizens [33]. The SPPB, standardized by the National Institute on Aging, includes three tests: balance, gait speed, and the five-times sit-to-stand (STS) test. The five-times STS test, conducted using a standardized armless chair (0.43 m in height), was used to calculate the STS mean muscle power (W; STS mean velocity × STS mean force) [34]. Dynamic balance was measured using the validated Timed Up and Go test (TUG) [35]. In addition, handgrip strength was assessed in the right arm with a Jamar hydraulic hand dynamometer according to a standardized protocol [36].

Habitual physical activity was measured using the Assessment of Physical Activity in Frail Older People (APAFOP) [37], an interview-administered questionnaire focusing on the activities typically performed in older age (e.g., walking, sitting, standing, lying). The APAFOP score was calculated by multiplying the total physical activity time during the previous 24 h by the metabolic equivalent of the task levels of the respective activities [38].

In order to ensure a more holistic assessment of frailty and given that each instrument regards frailty from different approaches, three measurement instruments were used so that frailty was measured using Fried’s frailty phenotype [39], the Rockwood Clinical Frailty Scale [40], and the Tilburg Frailty Indicator [41]. Fried’s frailty phenotype evaluates the presence of five criteria: slow walking speed, reduced grip strength, low physical activity, exhaustion, and unintentional weight loss. One point is given for each of the criteria and individuals scoring ≥ 3 are considered to be frail [39]. In the Clinical Frailty Scale, the frailty status is based on a clinical judgment; the scores range from 1 to 9 and individuals scoring ≥ 6 are considered to be frail [40]. The Tilburg Frailty Indicator contains 15 questions on physical, psychological, and social domains of frailty; the scores range from 0 to 9 and individuals scoring ≥ 5 are considered to be frail [41].

### 2.3. Statistical Analysis

A statistical analysis was performed using SPSS version 26.0. The continuous variables were expressed as a mean ± standard deviation (SD) and he categorical variables as frequencies and percentages. The normality was assessed using a Kolmogorov–Smirnov test and the non-parametric variables were square-root transformed. The group means were compared using Student´s *t*-tests. A series of linear regressions was used to separately analyze the relationships of functional status, physical fitness, physical activity, and frailty on the MNA, controlling for age and sex. The explanatory variables that were statistically significant in those regressions were included in a multiple linear regression model predicting the MNA, again controlling for age and sex. A stepwise backward elimination was used to select the final variables. The proportion of the variance in the MNA explained by the explanatory variables in the multiple regression was estimated by the coefficient of determination (R^2^). In all tests, the differences were considered significant at *p* < 0.05.

## 3. Results

Participant characteristics are summarized in Table 1.

**Figure 2:**
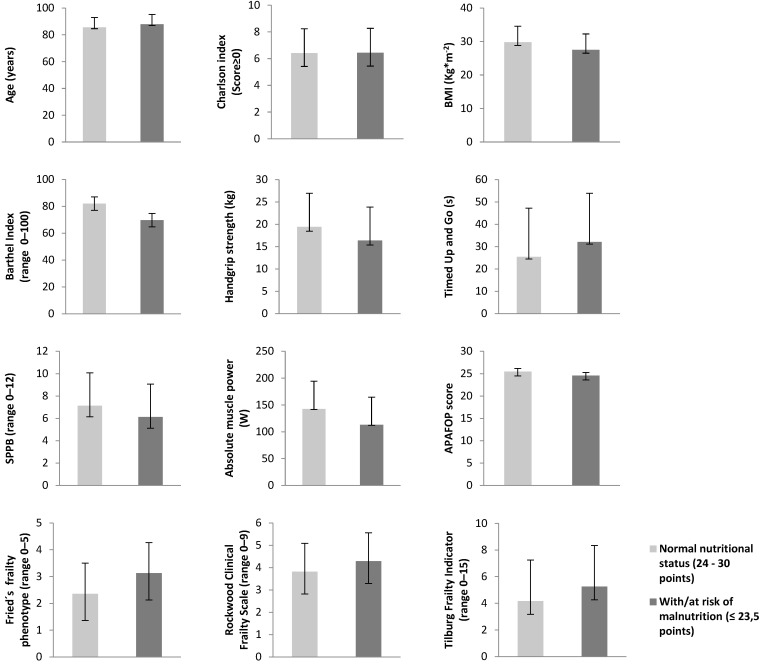
Characteristics of the participants according to the MNA classification (normal nutritional status vs. risk and malnutrition status). MNA: Mini Nutritional Assessment; BMI: body mass index; SPPB: Short Physical Performance Battery; APAFOP: Assessment of Physical Activity in Frail Older People. * *p* < 0.05 ** *p* < 0.01 *** *p* < 0.001.

Figure 2 summarizes the participant characteristics by the nutritional status (normal vs. malnutrition/risk of malnutrition). Compared with the participants with or at risk of malnutrition, those with a normal nutritional status had a higher BMI (*p* < 0.05), Barthel Index (*p* < 0.01), absolute muscle power (*p* < 0.05), and APAFOP score (*p* < 0.001) and a lower Fried’s frailty phenotype score (*p* < 0.01).

**Figure 3:**
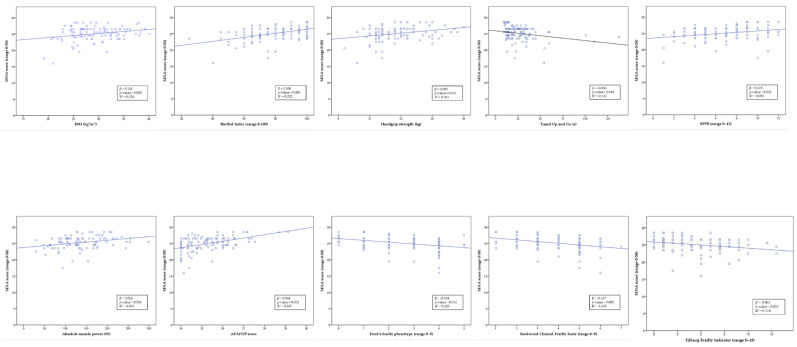
The relationship between the MNA score and functional status, physical performance, and physical activity, controlling for age and sex. BMI: body mass index; SPPB: Short Physical Performance Battery; APAFOP: Assessment of Physical Activity in Frail Older People. β: standardized regression coefficient; R^2^: coefficient of determination.

Figure 3 illustrates the relationship between the MNA scores and the parameters of functional status, physical performance, physical activity, and frailty. The MNA values were positively correlated with the Barthel Index score (*p* < 0.001), handgrip strength (*p* < 0.01), SPPB score (*p* < 0.01), absolute muscle power (*p* < 0.01), and APAFOP score (*p* < 0.001); they were negatively correlated with the TUG test score (*p* < 0.05), Fried’s frailty phenotype (*p* < 0.05), Rockwood Clinical Frailty Scale (*p* < 0.01), and Tilburg Frailty Indicator (*p* < 0.05) scores. The highest standardized regression coefficient (β) values were for the Barthel Index (β = 0.449) and APAFOP (β = 0.432) scores.

The explanatory variables included in the multiple linear regression model were the BMI, Barthel Index score, TUG score, SPPB score, handgrip strength, absolute muscle power, APAFOP score, and Fried’s frailty phenotype score (Table 2). Among these, the APAFOP score had the largest relationship (β = 0.386; *p* = 0.001), followed by the BMI (β = 0.301; *p* = 0.002) and the Barthel Index score (β = 0.220; *p* = 0.039). Together, these three variables explained 31% of the variance in the MNA.

## 4. Discussion

Despite the evidence that the nutritional status of older adults may affect functional and physical parameters, its effect on older adults living in LTNHs has remained unclear. Our results showed that a better nutritional status, as indicated by the higher MNA scores, was associated with greater functionality, physical performance, and physical activity as well as with lower frailty. In addition, physical activity (as measured by APAFOP questionnaire), functional status (as measured by the Barthel Index), and BMI collectively predicted the MNA. To our knowledge, this is the first study to find a relationship between the nutritional status and physical activity levels of LTNH residents without a cognitive impairment.

Our sample represented 1.75% of all older adults residing in LTNHs in Gipuzkoa (Basque Country, Northern Spain). Notably, our mean participant age, 86.34 ± 6.77 years, was higher than all other study populations to date on institutionalized older adults including the 2012 European SHELTER study of 57 LTNHs from 7 EU countries and Israel where the mean age of the participants was 83.4 years [42,43,44,45,46,47,48]. The percentage of participants in our study over 90 years of age (36%) was also high compared with other studies. Our population had several similarities with other studies. Most of the participants were women—a reflection of the LTNH resident population in general [42,43,45]—and about half were frail according to Fried’s frailty phenotype and the Tilburg Frailty Indicator, consistent with a systematic review on frailty prevalence in LTNHs [49]. In addition, a quarter of our participants were at risk of malnutrition (or actually malnourished), similar to the rates found in another recent study in institutionalized older adults [50].

From this perspective, the mean BMI value of our sample fell in the overweight range and was notably higher than in previous studies [42,43,46,51]. In addition, 42.1% were classified as overweight and 42.1% as obese. Growing evidence suggests that, in older individuals, a BMI between 25 and 30 that is defined as overweight may be a positive factor because it is associated with a longer life expectancy and especially with a longer disability-free life expectancy [51,52]. However, it should be emphasized that the BMI is a controversial measure as experts do not agree on its usefulness in predicting life expectancy in older adults [53]. Evidence for this is that obesity, in combination with sarcopenia or an age-related loss of skeletal muscle mass, increases the risk of cardiovascular disease, physical disability, metabolic disorders, and mortality [54]. Therefore, we recommend nutritional and physical activity interventions in older adults to try to preserve the muscle mass and prevent obesity.

LTNH residents are disproportionately at risk of malnutrition, potentially making them more likely to develop functional problems, sarcopenia, and frailty in the near future [55]. Our findings were consistent with previous works showing a link between malnutrition and functional dependence [43,44]. Consequently, to prevent malnutrition, a full understanding of the factors causing it is necessary.

Previous studies also found that physical performance is closely related to the nutritional status of institutionalized older adults and has an impact on their functionality, dependence level, and risk of falls [56,57]. In addition, an increased muscle strength reduces the risk of sarcopenia in older adults [58]. However, it is unclear whether a causal relationship exists between the nutritional status and physical performance. In a longitudinal study of community-dwelling older adults in Belgium, a three-year nutritional intervention did not affect handgrip strength or gait speed [59]. Further studies on this issue are, therefore, needed. In line with this and compared with the other parameters of physical performance, muscle power may be the capacity that is most related to functional limitations in older adults [60,61] including LTNH residents [62]. Given that few studies have related muscle power to nutrition [22,63], our results provide valuable evidence of this relationship and may inform new lines of research in which the variable of muscle power could be analyzed in order to ascertain the role that muscle power might play in the nutritional status of institutionalized older individuals. The link between physical activity and nutritional status is well-established as is the effect these two factors can have on the most prevalent problems in the older population such as sarcopenia, frailty, dependence, loss of functionality, and, ultimately, a decreased quality of life [64,65,66,67]. This is reflected in a recent meta-analysis where 26 different interventions were compared in older adults with sarcopenia using physical activity alone, nutrition alone, or a combination of both [68]. Exercise, both alone and in combination with nutrition, increased the handgrip strength and improved dynamic balance. Both types of intervention also had beneficial effects on muscle strength and the physical performance of older adults with sarcopenia [68].

The MNA provides important information for predicting frailty in older adults [69] and the relationship we found between nutrition and frailty, as measured by different tools, is well-established [24,25,70,71,72]. Nutritional frailty has been proposed to be an independent phenotype of frailty, underscoring the close relationship between these two variables [73]. The directionality of the relationships between nutritional status and functionality, physical performance, physical activity, and frailty is uncertain. Based on the results of the present study, it could be intuitively deduced that a few of these relationships may be bidirectional. A multicomponent physical activity intervention in older adults after hospitalization improved the nutritional status of participants [74]. It has also been observed that a functional evolution through a rehabilitation process was impaired in orthogeriatric patients with malnutrition [56].

In order to delve deeper into this bidirectional relationship and draw conclusions about causality, intervention programs are needed. To our knowledge, few intervention programs in institutionalized older populations have measured both the nutritional parameters and physical activity [75,76,77,78,79]. Two studies on the effects of a physical activity intervention on the nutritional status found no significant changes [76,77]. This lack of favorable results could be due to the cognitive characteristics of the participants (e.g., Alzheimer’s disease), a low adherence to the program, or insufficient exercise intensity. For example, although the Report of the International Sarcopenia Initiative recommends supervised resistance exercise for individuals with sarcopenia [71], the participants in those studies did not use weights. On the other hand, nutritional interventions [79,80], or those combining nutrition and physical activity [75], can improve the functional as well as the nutritional parameters. In addition, essential amino acids and β-Hydroxy β-methylbutyric acid (HMB) supplements may improve the muscle outcomes [71].

Different studies have concluded that both nutrition and physical activity improve the functional status of, and prevent sarcopenia in, older adults [81,82,83]. Nutritional supplementation may improve the muscle status or, conversely, muscle work/training may improve the nutritional status, perhaps by stimulating the appetite and thus increasing the caloric intake. Both muscle and visceral protein levels were found to decrease with age among LTNH residents, leading to a higher prevalence of malnutrition [84]. This decreased muscle mass depended in part on the physical activity levels of participants as measured by the ADL. Thus, physical activity can play an important role in the nutritional status of institutionalized older individuals.

Although our study did not address causality, it did support the hypothesis that physical activity may possibly improve the nutritional status of institutionalized older adults [26,85]. Therefore, strategies to increase physical activity may help to promote a better quality of life among residents of nursing homes.

Taking into account these data and given the close relationship we found between the MNA and functional status, physical performance and activity, and frailty, future approaches to improve these parameters in LTNH residents should not be focused on each variable individually but on all these variables together. Interdisciplinary professional teams (doctors, nurses, physiotherapists, nutritionists, physical activity professionals) should participate in the design and implementation of strategies to simultaneously improve the nutritional and functional status and physical performance of LTNH residents to increase health and the quality of life. Our results reinforce the need to develop updated physical activity and nutrition routines in LTNHs and may play a fundamental role in the redesign of nursing homes for the better overall wellness of the residents.

Finally, and based on our results, we consider it essential to implement preventive actions that allow the diagnosis and treatment as early as possible of the appearance of pathologies that directly affect the quality of life of LTNH residents. In the future, and with the aim of being able to analyze the causality that we hypothesized in the present study, it is necessary to develop further nutritional and physical activity interventions.

## 5. Strength of the Study

One of the strengths of this study is the combined analysis of the relationships between nutritional status and functional status, physical performance, physical activity and frailty. To our knowledge, this is the first study on LTNH residents without a cognitive impairment to relate nutritional status with physical activity. Our results may help inform the design of future studies.

## 6. Limitations of the Study

Our study also had several limitations. Due to the difficulty in precisely measuring the body composition of an institutionalized population, we used the BMI, a relatively simple measurement. Analyzing the fat mass and skeletal muscle mass would provide more detailed information on the relationship between nutritional status and the measurements indicative of sarcopenia. Similarly, although the MNA is validated and widely used in the LTNH environment [86], a more detailed analysis of the food and caloric intake would shed further light on the relationship between nutritional status and physical activity among institutionalized older adults.

## 7. Conclusions

Our study demonstrated that the nutritional status measured by the MNA was related to functional status, physical fitness and activity, and frailty in older adults living in LTNHs. The results of our study highlighted the importance of taking into account all these parameters as a whole in order to maintain and care for the quality of life of older adults living in LTNHs.

## Figures and Tables

**Table 1:** Participant characteristics.

	Total Sample, *n* = 105
Women, *n* (%)	57 (54.3)
Men, *n* (%)	48 (45.7)
Age, *n* (%)	
70–80 years	23 (21.9)
80–90 years	46 (43.8)
>90 years	36 (34.3)
MNA, *n* (%)	
Normal nutritional status	78 (74.2)
Risk of malnutrition	26 (24.8)
Malnutrition	1 (1.0)
Charlson index (score ≥ 0)	6.42 ± 1.86
BMI (kg·m^−2^)	29.28 ± 4.60
Normal weight, *n* (%)	17 (16.7)
Overweight, *n* (%)	42 (41.2)
Obesity, *n* (%)	42 (41.2)
Morbid obesity, *n* (%)	1 (1)
Barthel Index (range 0–100)	79.17 ± 16.61
Handgrip strength (Kg)	18.72 ± 7.61
Timed Up and Go (s)	27.05 ± 19.75
SPPB (range 0–12)	6.90 ± 2.86
Absolute muscle power (W)	149.78 ± 72.11
APAFOP score	25.29 ± 1.09
Fried’s frailty phenotype (range 0–5)	2.54 ± 1.38
Not frail, *n* (%)	45 (42.9)
Frail, *n* (%)	60 (57.1)
Rockwood Clinical Frailty Scale (range 0–9)	3.93 ± 1.41
Not frail, *n* (%)	92 (87.6)
Frail, *n* (%)	13 (12.4)
Tilburg Frailty Indicator (range 0–15)	4.42 ± 3.16
Not frail, *n* (%)	55 (52.4)
Frail, *n* (%)	50 (47.6)

MNA: Mini Nutritional Assessment; BMI: body mass index; SPPB: Short Physical Performance Battery; APAFOP: Assessment of Physical Activity in Frail Older People.

**Table 2:** Backward stepwise multiple linear regression model predicting the MNA score, controlling for age and sex.

	β	Standardized β	*p*-Value	R^2^
Intercept	0.003		0.998	0.314
BMI (kg·m^−2^)	0.150	0.301	0.002	
Barthel Index score	0.050	0.220	0.039	
APAFOP score	0.746	0.386	0.001	

BMI: body mass index; APAFOP: Assessment of Physical Activity in Frail Older People.

## Data Availability

The data presented in this study are available on request from the corresponding author.

## References

[1] Salva A., Coll-Planas L., Bruce S., De Groot L., Andrieu S., Abellan G., Vellas B., The Task Force on Nutrition and Ageing of the IAGG and the IANA (2009). Nutritional Assessment of Residents in Long-Term Care Facilities (LTCFs): Recommendations of the Task Force on Nutrition and Ageing of the IAGG European Region and the IANA. J. Nutr. Health Aging.

[2] Mathewson S.L., Azevedo P.S., Gordon A.L., Phillips B.E., Greig C.A. (2021). Overcoming protein-energy malnutrition in older adults in the residential care setting: A narrative review of causes and interventions. Ageing Res. Rev..

[3] Clegg M.E., Williams E.A. (2018). Optimizing nutrition in older people. Maturitas.

[4] Brownie S. (2006). Why are elderly individuals at risk of nutritional deficiency?. Int. J. Nurs. Pract..

[5] Kiesswetter E., Colombo M.G., Meisinger C., Peters A., Thorand B., Holle R., Ladwig K.H., Schulz H., Grill E., Diekmann R. (2020). Malnutrition and Related Risk Factors in Older Adults from Different Health-Care Settings: An Enable Study. Public Health Nutr..

[6] Roberts C.E., Phillips L.H., Cooper C.L., Gray S., Allan J.L. (2017). Effect of Different Types of Physical Activity on Activities of Daily Living in Older Adults: Systematic Review and Meta-Analysis. J. Aging Phys. Act..

[7] Motamed-Jahromi M., Kaveh M.H. (2021). Effective Interventions on Improving Elderly’s Independence in Activity of Daily Living: A Systematic Review and Logic Model. Front. Public Health.

[8] Glass N.L., Bellettiere J., Jain P., LaMonte M.J., La Croix A.Z., Women’s Health Initiative (2021). Evaluation of Light Physical Activity Measured by Accelerometry and Mobility Disability during a 6-Year Follow-Up in Older Women. JAMA Netw. Open.

[9] Stattin K., Michaelsson K., Larsson S.C., Wolk A., Byberg L. (2017). Leisure-Time Physical Activity and Risk of Fracture: A Cohort Study of 66,940 Men and Women. J. Bone Miner. Res..

[10] Kok R.M., Reynolds C.F. (2017). Management of Depression in Older Adults: A Review. JAMA.

[11] Murri M.B., Ekkekakis P., Menchetti M., Neviani F., Trevisani F., Tedeschi S., Latessa P.M., Nerozzi E., Ermini G., Zocchi D. (2018). Physical Exercise for Late-Life Depression: Effects on Symptom Dimensions and Time Course. J. Affect. Disord..

[12] Moraes H.S., Silveira H.S., Oliveira N.A., Matta Mello Portugal E., Araujo N.B., Vasques P.E., Bergland A., Santos T.M., Engedal K., Coutinho E.S. (2020). Is Strength Training as Effective as Aerobic Training for Depression in Older Adults? A Randomized Controlled Trial. Neuropsychobiology.

[13] Ng T.P., Feng L., Nyunt M.S., Feng L., Niti M., Tan B.Y., Chan G., Khoo S.A., Chan S.M., Yap P. (2015). Nutritional, Physical, Cognitive, and Combination Interventions and Frailty Reversal among Older Adults: A Randomized Controlled Trial. Am. J. Med..

[14] Tarazona-Santabalbina F.J., Gomez-Cabrera M.C., Perez-Ros P., Martinez-Arnau F.M., Cabo H., Tsaparas K., Salvador-Pascual A., Rodriguez-Manas L., Vina J. (2016). A Multicomponent Exercise Intervention that Reverses Frailty and Improves Cognition, Emotion, and Social Networking in the Community-Dwelling Frail Elderly: A Randomized Clinical Trial. J. Am. Med. Dir. Assoc..

[15] Arrieta H., Rezola-Pardo C., Echeverria I., Iturburu M., Gil S.M., Yanguas J.J., Irazusta J., Rodriguez-Larrad A. (2018). Physical Activity and Fitness are Associated with Verbal Memory, Quality of Life and Depression among Nursing Home Residents: Preliminary Data of a Randomized Controlled Trial. BMC Geriatr..

[16] Saghafi-Asl M., Vaghef-Mehrabany E. (2017). Comprehensive Comparison of Malnutrition and its Associated Factors between Nursing Home and Community Dwelling Elderly: A Case-Control Study from Northwestern Iran. Clin. Nutr. ESPEN.

[17] Tay L.B., Chua M.P., Tay E.L., Chan H.N., Mah S.M., Latib A., Wong C.Q., Ng Y.S. (2019). Multidomain Geriatric Screen and Physical Fitness Assessment Identify Prefrailty/Frailty and Potentially Modifiable Risk Factors in Community-Dwelling Older Adults. Ann. Acad. Med. Singap..

[18] Aspell N., Laird E., Healy M., Lawlor B., O’Sullivan M. (2019). Vitamin D Deficiency is Associated with Impaired Muscle Strength and Physical Performance in Community-Dwelling Older Adults: Findings from the English Longitudinal Study of Ageing. Clin. Interv. Aging.

[19] Ge L., Yap C.W., Heng B.H. (2020). Association of Nutritional Status with Physical Function and Disability in Community-Dwelling Older Adults: A Longitudinal Data Analysis. J. Nutr. Gerontol. Geriatr..

[20] Izawa S., Enoki H., Hasegawa J., Hirose T., Kuzuya M. (2014). Factors Associated with Deterioration of Mini Nutritional Assessment-Short Form Status of Nursing Home Residents during a 2-Year Period. J. Nutr. Health Aging.

[21] Vandewoude M.F.J., van Wijngaarden J.P., De Maesschalck L., Luiking Y.C., Van Gossum A. (2019). The Prevalence and Health Burden of Malnutrition in Belgian Older People in the Community or Residing in Nursing Homes: Results of the NutriAction II Study. Aging Clin. Exp. Res..

[22] Ramsey K.A., Meskers C.G.M., Trappenburg M.C., Verlaan S., Reijnierse E.M., Whittaker A.C., Maier A.B. (2020). Malnutrition is Associated with Dynamic Physical Performance. Aging Clin. Exp. Res..

[23] Corcoran M.P., Nelson M.E., Sacheck J.M., Reid K.F., Kirn D., Fielding R.A., Chui K.K.H., Folta S.C. (2017). Efficacy of an Exercise and Nutritional Supplement Program on Physical Performance and Nutritional Status in Older Adults with Mobility Limitations Residing at Senior Living Facilities. J. Aging Phys. Act..

[24] Hernandez Morante J.J., Gomez Martinez C., Morillas-Ruiz J.M. (2019). Dietary Factors Associated with Frailty in Old Adults: A Review of Nutritional Interventions to Prevent Frailty Development. Nutrients.

[25] Ni Lochlainn M., Cox N.J., Wilson T., Hayhoe R.P.G., Ramsay S.E., Granic A., Isanejad M., Roberts H.C., Wilson D., Welch C. (2021). Nutrition and Frailty: Opportunities for Prevention and Treatment. Nutrients.

[26] Fiatarone Singh M.A., Bernstein M.A., Ryan A.D., O’Neill E.F., Clements K.M., Evans W.J. (2000). The effect of oral nutritional supplements on habitual dietary quality and quantity in frail elders. J. Nutr. Health Aging.

[27] Smoliner C., Norman K., Scheufele R., Hartig W., Pirlich M., Lochs H. (2008). Effects of Food Fortification on Nutritional and Functional Status in Frail Elderly Nursing Home Residents at Risk of Malnutrition. Nutrition.

[28] Lobo A., Saz P., Marcos G., Día J.L., de la Cámara C., Ventura T., Morales Asín F., Fernando Pascual L., Montañés J.Á., Aznar S. (1999). Revalidación Y Normalización Del Mini-Examen Cognoscitivo (Primera Versión En Castellano Del Mini-Mental Status Examination) En La Población General Geriátrica. Med. Clin..

[29] Guigoz Y., Vellas B., Garry P.J. (1996). Assessing the Nutritional Status of the Elderly: The Mini Nutritional Assessment as Part of the Geriatric Evaluation. Nutr. Rev..

[30] Charlson M., Szatrowski T.P., Peterson J., Gold J. (1994). Validation of a Combined Comorbidity Index. J. Clin. Epidemiol..

[31] NHLBI Obesity Education Initiative Expert Panel on the Identification, Evaluation, and Treatment of Obesity in Adults (US) (1998). Clinical Guidelines on the Identification, Evaluation, and Treatment of Overweight and Obesity in Adults: The Evidence Report.

[32] Wade D.T., Collin C. (1988). The Barthel ADL Index: A Standard Measure of Physical Disability?. Int. Disabil. Stud..

[33] Guralnik J.M., Ferrucci L., Pieper C.F., Leveille S.G., Markides K.S., Ostir G.V., Wallace R.B. (2000). Lower extremity function and subsequent disability: Consistency across studies, predictive models, and value of gait speed alone compared with the short physical performance battery. J. Gerontol. Ser. A.

[34] Alcazar J., Losa-Reyna J., Rodriguez-Lopez C., Alfaro-Acha A., Rodriguez-Mañas L., Ara I., García-García F.J., Alegre L.M. (2018). The Sit-to-Stand Muscle Power Test: An Easy, Inexpensive and Portable Procedure to Assess Muscle Power in Older People. Exp. Gerontol..

[35] Bohannon R.W. (2006). Reference Values for the Timed Up and Go Test: A Descriptive Meta-Analysis. J. Geriatr. Phys. Ther..

[36] Fess E. (1981). Clinical Assessment Recommendations. American society of hand therapists. Grip Strength.

[37] Hauer K., Lord S.R., Lindemann U., Lamb S.E., Aminian K., Schwenk M. (2011). Assessment of Physical Activity in Older People with and without Cognitive Impairment. J. Aging Phys. Act..

[38] Ainsworth B.E., Haskell W.L., Whitt M.C., Irwin M.L., Swartz A.M., Strath S.J., Brien W.L.O., Bassett D.R., Schmitz K.H., Emplaincourt P.O. (2000). Compendium of Physical Activities: An Update of Activity Codes and MET Intensities. Med. Sci. Sports Exerc..

[39] Fried L.P., Tangen C.M., Walston J., Newman A.B., Hirsch C., Gottdiener J., Seeman T., Tracy R., Kop W.J., Burke G. (2001). Frailty in Older Adults: Evidence for a Phenotype. J. Gerontol. Ser. A Biol. Sci. Med Sci..

[40] Rockwood K., Theou O. (2020). Using the Clinical Frailty Scale in Allocating Scarce Health Care Resources. Can. Geriatr. J..

[41] Gobbens R.J., van Assen M.A., Luijkx K.G., Wijnen-Sponselee M.T., Schols J.M. (2010). The Tilburg Frailty Indicator: Psychometric Properties. J. Am. Med. Dir. Assoc..

[42] Piglowska M., Guligowska A., Kostka T. (2020). Nutritional Status Plays More Important Role in Determining Functional State in Older People Living in the Community than in Nursing Home Residents. Nutrients.

[43] Caçador C., Teixeira-Lemos E., Oliveira J., Pinheiro J., Mascarenhas-Melo F., Ramos F. (2021). The Relationship between Nutritional Status and Functional Capacity: A Contribution Study in Institutionalised Portuguese Older Adults. Int. J. Environ. Res. Public Health.

[44] Donini L.M., Stephan B., Rosano A., Molfino A., Poggiogalle E., Lenzi A., Siervo M., Muscaritoli M. (2020). What are the Risk Factors for Malnutrition in Older-Aged Institutionalized Adults?. Nutrients.

[45] Jerez-Roig J., de Brito Macedo Ferreira L.M., Torres de Araújo J.R., Costa Lima K. (2017). Functional Decline in Nursing Home Residents: A Prognostic Study. PLoS ONE.

[46] López-Contreras M., Torralba C., Zamora S., Pérez-Llamas F. (2012). Nutrition and Prevalence of Undernutrition Assessed by Different Diagnostic Criteria in Nursing Homes for Elderly People. J. Hum. Nutr. Diet..

[47] de Medeiros M.M.D., de Figueredo O.M.C., Pinheiro M.A., de Oliveira L.F.S., Wanderley R.L., Cavalcanti Y.W., Garcia R.C.M.R. (2020). Factors Associated with the Overlap of Frailty and Nutrition in Institutionalized Older Adults: A Multicenter Study. Arch. Gerontol. Geriatr..

[48] Onder G., Carpenter I., Finne-Soveri H., Gindin J., Frijters D., Henrard J.C., Nikolaus T., Topinkova E., Tosato M., Liperoti R. (2012). Assessment of Nursing Home Residents in Europe: The Services and Health for Elderly in Long TERm Care (SHELTER) Study. BMC Health Serv. Res..

[49] Kojima G. (2015). Prevalence of Frailty in Nursing Homes: A Systematic Review and Meta-Analysis. J. Am. Med. Dir. Assoc..

[50] Faxen-Irving G., Luiking Y., Gronstedt H., Franzen E., Seiger A., Vikstrom S., Wimo A., Bostrom A.M., Cederholm T. (2021). Do Malnutrition, Sarcopenia and Frailty Overlap in Nursing-Home Residents?. J. Frailty Aging.

[51] Al Snih S., Ottenbacher K.J., Markides K.S., Kuo Y., Eschbach K., Goodwin J.S. (2007). The Effect of Obesity on Disability Vs Mortality in Older Americans. Arch. Intern. Med..

[52] Winter J.E., MacInnis R.J., Wattanapenpaiboon N., Nowson C.A. (2014). BMI and all-Cause Mortality in Older Adults: A Meta-Analysis. Am. J. Clin. Nutr..

[53] Bosello O., Vanzo A. (2021). Obesity Paradox and Aging. Eat. Weight Disord..

[54] Atkins J.L., Wannamathee S.G. (2020). Sarcopenic Obesity in Ageing: Cardiovascular Outcomes and Mortality. Br. J. Nutr..

[55] Liu W., Chen S., Jiang F., Zhou C., Tang S. (2020). Malnutrition and Physical Frailty among Nursing Home Residents: A Cross-Sectional Study in China. J. Nutr. Health Aging.

[56] Urquiza M., Fernandez N., Arrinda I., Sierra I., Irazusta J., Rodriguez Larrad A. (2020). Nutritional Status is Associated with Function, Physical Performance and Falls in Older Adults Admitted to Geriatric Rehabilitation: A Retrospective Cohort Study. Nutrients.

[57] Aparicio-Ugarriza R., Luzardo-Socorro R., Palacios G., Bibiloni M.M., Argelich E., Tur J.A., Gonzalez-Gross M. (2019). What is the Relationship between Physical Fitness Level and Macro- and Micronutrient Intake in Spanish Older Adults?. Eur. J. Nutr..

[58] Cruz-Jentoft A.J., Bahat G., Bauer J., Boirie Y., Bruyere O., Cholm T., Cooper C., Landi F., Rolland Y., Sayer A.A. (2019). Sarcopenia: Revised European Consensus on Definition and Diagnosis. Age Ageing.

[59] Lengele L., Moehlinger P., Bruyere O., Locquet M., Reginster J.Y., Beaudart C. (2020). Association between Changes in Nutrient Intake and Changes in Muscle Strength and Physical Performance in the SarcoPhAge Cohort. Nutrients.

[60] Martinikorena I., Martinez-Ramirez A., Gomez M., Lecumberri P., Casas-Herrero A., Cadore E.L., Millor N., Zambom-Ferraresi F., Idoate F., Izquierdo M. (2016). Gait Variability Related to Muscle Quality and Muscle Power Output in Frail Nonagenarian Older Adults. J. Am. Med. Dir. Assoc..

[61] Foldvari M., Clark M., Laviolette L.C., Bernstein M.A., Kaliton D., Castaneda C., Pu C.T., Hausdorff J.M., Fielding R.A., Singh M.A.F. (2000). Association of Muscle Power with Functional Status in Community-Dwelling Elderly Women. J. Gerontol. Ser. A Biol. Sci. Med. Sci..

[62] Kozicka I., Kostka T. (2016). Handgrip Strength, Quadriceps Muscle Power, and Optimal Shortening Velocity Roles in Maintaining Functional Abilities in Older Adults Living in a Long-Term Care Home: A 1-Year Follow-Up Study. Clin. Interv. Aging.

[63] Bonnefoy M., Jauffret M., Jusot J.F. (2007). Muscle Power of Lower Extremities in Relation to Functional Ability and Nutritional Status in very Elderly People. J. Nutr. Health Aging.

[64] Abizanda P., Lopez M.D., Garcia V.P., Estrella Jde D., da Silva Gonzalez A., Vilardell N.B., Torres K.A. (2015). Effects of an Oral Nutritional Supplementation Plus Physical Exercise Intervention on the Physical Function, Nutritional Status, and Quality of Life in Frail Institutionalized Older Adults: The ACTIVNES Study. J. Am. Med. Dir. Assoc..

[65] Phu S., Boersma D., Duque G. (2015). Exercise and Sarcopenia. J. Clin. Densitom..

[66] Rus G.E., Porter J., Brunton A., Crocker M., Kotsimbos Z., Percic J., Polzella L., Willet N., Huggins C.E. (2020). Nutrition Interventions Implemented in Hospital to Lower Risk of Sarcopenia in Older Adults: A Systematic Review of Randomised Controlled Trials. Nutr. Diet..

[67] Marshall R.N., Smeuninx B., Morgan P.T., Breen L. (2020). Nutritional Strategies to Offset Disuse-Induced Skeletal Muscle Atrophy and Anabolic Resistance in Older Adults: From Whole-Foods to Isolated Ingredients. Nutrients.

[68] Wu P.Y., Huang K.S., Chen K.M., Chou C.P., Tu Y.K. (2021). Exercise, Nutrition, and Combined Exercise and Nutrition in Older Adults with Sarcopenia: A Systematic Review and Network Meta-Analysis. Maturitas.

[69] Valentini A., Federici M., Cianfarani M.A., Tarantino U., Bertoli A. (2018). Frailty and Nutritional Status in Older People: The Mini Nutritional Assessment as a Screening Tool for the Identification of Frail Subjects. Clin. Interv. Aging.

[70] Artaza-Artabe I., Saez-Lopez P., Sanchez-Hernandez N., Fernandez-Gutierrez N., Malafarina V. (2016). The Relationship between Nutrition and Frailty: Effects of Protein Intake, Nutritional Supplementation, Vitamin D and Exercise on Muscle Metabolism in the Elderly. A Systematic Review. Maturitas.

[71] Cruz-Jentoft A.J., Landi F., Schneider S.M., Zuniga C., Arai H., Boirie Y., Chen L.K., Fielding R.A., Martin F.C., Michel J.P. (2014). Prevalence of and Interventions for Sarcopenia in Ageing Adults: A Systematic Review. Report of the International Sarcopenia Initiative (EWGSOP and IWGS). Age Ageing.

[72] Lorenzo-Lopez L., Maseda A., de Labra C., Regueiro-Folgueira L., Rodriguez-Villamil J.L., Millan-Calenti J.C. (2017). Nutritional Determinants of Frailty in Older Adults: A Systematic Review. BMC Geriatr..

[73] Zupo R., Castellana F., Bortone I., Griseta C., Sardone R., Lampignano L., Lozupone M., Solfrizzi V., Castellana M., Giannelli G. (2020). Nutritional Domains in Frailty Tools: Working Towards an Operational Definition of Nutritional Frailty. Ageing Res. Rev..

[74] Echeverria I., Amasene M., Urquiza M., Labayen I., Anaut P., Rodriguez-Larrad A., Irazusta J., Besga A. (2020). Multicomponent Physical Exercise in Older Adults After Hospitalization: A Randomized Controlled Trial Comparing Short- Vs. Long-Term Group-Based Interventions. Int. J. Environ. Res. Public. Health..

[75] Beck A.M., Christensen A.G., Hansen B.S., Damsbo-Svendsen S., Moller T.K. (2016). Multidisciplinary Nutritional Support for Undernutrition in Nursing Home and Home-Care: A Cluster Randomized Controlled Trial. Nutrition.

[76] Rolland Y., Pillard F., Klapouszczak A., Reynish E., Thomas D., Andrieu S., Riviere D., Vellas B. (2007). Exercise Program for Nursing Home Residents with Alzheimer’s Disease: A 1-Year Randomized, Controlled Trial. J. Am. Geriatr. Soc..

[77] Maltais M., Rolland Y., Hay P.E., Armaingaud D., Cestac P., Rouch L., de Souto Barreto P. (2018). The Effect of Exercise and Social Activity Interventions on Nutritional Status in Older Adults with Dementia Living in Nursing Homes: A Randomised Controlled Trial. J. Nutr. Health Aging.

[78] Senior H.E., Henwood T.R., Beller E.M., Mitchell G.K., Keogh J.W. (2015). Prevalence and Risk Factors of Sarcopenia among Adults Living in Nursing Homes. Maturitas.

[79] Christensson L., Ek A.C., Unosson M. (2001). Individually Adjusted Meals for Older People with Protein-Energy Malnutrition: A Single-Case Study. J. Clin. Nurs..

[80] Allen V.J., Methven L., Gosney M.A. (2013). Use of Nutritional Complete Supplements in Older Adults with Dementia: Systematic Review and Meta-Analysis of Clinical Outcomes. Clin. Nutr..

[81] Papadopoulou S.K. (2020). Sarcopenia: A Contemporary Health Problem among Older Adult Populations. Nutrients.

[82] Franzke B., Neubauer O., Cameron-Smith D., Wagner K.H. (2018). Dietary Protein, Muscle and Physical Function in the very Old. Nutrients.

[83] Bosaeus I., Rothenberg E. (2016). Nutrition and Physical Activity for the Prevention and Treatment of Age-Related Sarcopenia. Proc. Nutr. Soc..

[84] Nakamura H., Fukushima H., Miwa Y., Shiraki M., Gomi I., Saito M., Mawatari K., Kobayashi H., Kato M., Moriwaki H. (2006). A Longitudinal Study on the Nutritional State of Elderly Women at a Nursing Home in Japan. Intern. Med..

[85] Poehlman E.T., Dvorak R.V. (2000). Energy Expenditure, Energy Intake, and Weight Loss in Alzheimer Disease. Am. J. Clin. Nutr..

[86] Diekmann R., Winning K., Uter W., Kaiser M.J., Sieber C.C., Volkert D., Bauer J.M. (2013). Screening for Malnutrition among Nursing Home Residents—A Comparative Analysis of the Mini Nutritional Assessment, the Nutritional Risk Screening, and the Malnutrition Universal Screening Tool. J. Nutr. Health Aging.

